# Highly Responsive Blue Light Sensor with Amorphous Indium-Zinc-Oxide Thin-Film Transistor based Architecture

**DOI:** 10.1038/s41598-018-26580-5

**Published:** 2018-05-25

**Authors:** Po Tsun Liu, Dun Bao Ruan, Xiu Yun Yeh, Yu Chuan Chiu, Guang Ting Zheng, Simon M. Sze

**Affiliations:** 1 Department of Photonics and Institute of Electro-Optical Engineering, National Chiao Tung University, Hsinchu, 30010 Taiwan; 2 Department of Electronics Engineering, National Chiao Tung University, Hsinchu, 30010 Taiwan

## Abstract

A single layer of amorphous InZnO is chosen as the channel material for a thin film transistor (TFT)-based driver and sensing layer for a blue-light sensor, respectively, with a completely compatible process integrated into in-cell embedded photo sensor architecture. The photo sensor exhibits a high optical responsivity (1280 A/W) and excellent signal to noise ratio (~10^5^) under the blue light illumination. Afterwards, the detail studies and important issues about the sensing and material characteristics of a-IZO thin film in the TFT sensor are well discussed. The results suggest that the numbers of the deep, neutral oxygen vacancy are the key factors for carrier generation under illumination. In addition, a positive gate pulse is applied on the devices to eliminate persistent photoconductivity in order to ensure the recover ability for the photo sensor application. The practical concepts of a sensor circuit, which can be integrated on RGB pixel with interactive display, are also proposed on the basis of photo sensor TFT.

## Introduction

With the rapid progress and continuing improvements of thin-film transistors (TFTs) technologies over the past few years, TFT display panels have been developed not only for screens but also for other applications, like flexible electronics, biomedicine sensors, nonvolatile memories, 3-dimentional integrated circuits^1–5^. Among those novel applications, the most interesting topic of TFT-LCDs would be the optical detection sensor. The sensor can be integrated into display panels of hand-held consumer products for realizing photo-sensing functions such as ambient light sensors, image scanners, touch panel, etc^6–11^. Besides, amorphous metal oxide (AMO), especially the amorphous InZnO (a-IZO), has attracted much attention as the active layer material recently^12^. Unlike the conventional amorphous silicon (a-Si) TFT with poor carrier transport properties or the polycrystalline silicon (poly-Si) TFTs with poor uniformity over a large area, a-IZO shows a high electron mobility (~10 cm^2^/V.s), good uniformity, low manufacturing cost and low process temperature^13,14^. Furthermore, the band-gap energy of IZO is about 2.9 eV, which means that the blue light with a wavelength of 430 nm can be absorbed or detected by this material^15^. Additionally, there are more sub-gap states located 2 eV below the mobility edge of IZO at deep state, compared with the traditional amorphous InGaZnO (a-IGZO) channel material. This means there is a higher probability for ionizing the deep, neutral oxygen vacancy states (V_O_) to the shallow donor states (V_O_^2+^) during the light illumination in IZO^16^. Therefore, the optical responsivity and signal to noise ratio can be further improved by just using a single layer a-IZO channel material, especially under the blue light. Coincidentally, unlike many research works exploring the full spectrum light sensing, the literatures focusing on the blue-light sensing combined with a-IZO TFT technology and an exhaustive discussion about the blue-light sensor device characteristics and operation mechanisms are still seldom in the academic.

In particular, a high performance blue-light sensor shows its high commercial potentiality for many future scientific applications, like self-piloting automobile under serious smog environment, fingerprint or precision image scanning, illumination self-adjustment of smart agriculture and biomedical electronic research, which are summarized in Figure S1 of Supplementary. In detail, the technology of light detection and ranging (LIDAR) is widely used in the driverless vehicles. However, it may lose efficacy under serious smog ambient which will cause severe problem of safety control. Hence, a blue-light sensor with high optical responsivity and signal to noise ratio may be the final solution for its good anti-jamming performances within the smog compared with other frequencies of illumination. Similarly, the optical sensor in the blue-visible sensing region can also be utilized in the name card, fingerprint or precision image scanning for its good photo sensing characteristics in terms of high quantum efficiency, optical responsivity, signal to noise ratio, and reliability^17–19^. On the other hand, blue light is especially effective in photomorphogenic responses through the action of blue/UV-A photoreceptor(s), and UV-B photoreceptor(s)^20^. Chlorophyll has strong absorption close to wavelength of blue light for the photosynthesis^21^. In the concept of smart agriculture, the dosage of blue light needs to be controlled by a good photo sensor device for increasing the yield of crops, which can be named as illumination self-adjustment system. In addition, blue light illumination is also classified as a “photoreceptor damage” for human being. There are much experimental evidence indicated that blue-light exposure can affect many physiologic functions^22–25^. Some researchers have shown that the blue light could induce oxidative stress preferentially in mitochondria of live skin^22^. With exposing human skin to the blue light, the flavin autofluorescence depressed, which indicates that the visible component of blue light has a physiologically significant effect on human skin^23^. Not only that, but several studies have reported that blue light is more effective in melanopsin secretion which may affect sleep and human circadian clock^24^. Besides, numerous studies confirm that cumulative lifetime exposure to blue light causes photo-oxidation of retinal cells^25^. Therefore, a lot of attentions need to be paid on the blue-light sensor exploitation.

In this work, a single layer of amorphous In-Zn-O thin film is chosen as the active layer material for both the drive device channel and blue-light sensing function. Meanwhile, with the detail material analysis and physical model, the photo sensing characteristics, channel length effect, and persistent photoconductivity of a-IZO sensor TFT are also well discussed under blue light illumination. Beside, other important issues about this sensor TFT architecture like the reliability of driver section, temperature effect of sensor section, and the uniformity characteristics are also included in this work (Supplementary). Furthermore, an embedded photo sensor with in-cell structure and a sensor circuit model of the conventional bottom-gate drive TFT and sensor TFT, which can be integrated by a compatible process on the same Si substrate, are also proposed.

## Results

**Figure 1:**
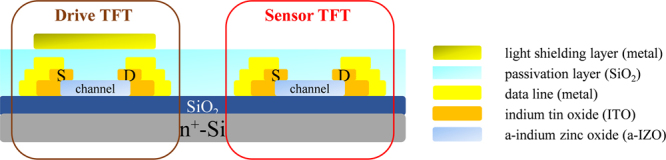
The cross-sectional view of a-IZO TFT with (drive TFT) and without (sensor TFT) a top light blocking metal layer embedded in touch display panel structure in this work.

Figure 1 shows the cross-sectional view of an in-cell structure with sensor TFT and drive TFT embedded in a display panel in this work. The pixel of the interactive panel will consist of both RGB display section and sensing section with this in-cell structure. Unlike the traditional discrete add-on optical photo sensor integrated by post-process or system-in-package technology, which may increase the volume of display module, decreasing the light transmittance, lowering sensor sensitivity and display resolution, there are some more benefits for these embedded in-cell structures without the problem above-mentioned^26,27^. One merit is that each pixel or group of pixels could be individually detectable, which may suppress the loss of the photo sensor sensitivity caused by the transportation of the light signal between the sensing circuit through additional sensing panel. Another benefit of embedded in-cell structure is the utilization of the same process technology for both drive TFT and sensor TFT on the same substrate just divided by a top light blocking metal layer. In addition, the embedded in-cell architecture can also achieve many complicated circuit modules more easily than the add-on technology, for example, like some compensation circuit models to decreasing the influence of uniformity problem and temperature effect, or a signal amplifier to filter and select recovered signal of persistent photoconductivity.

**Figure 2:**
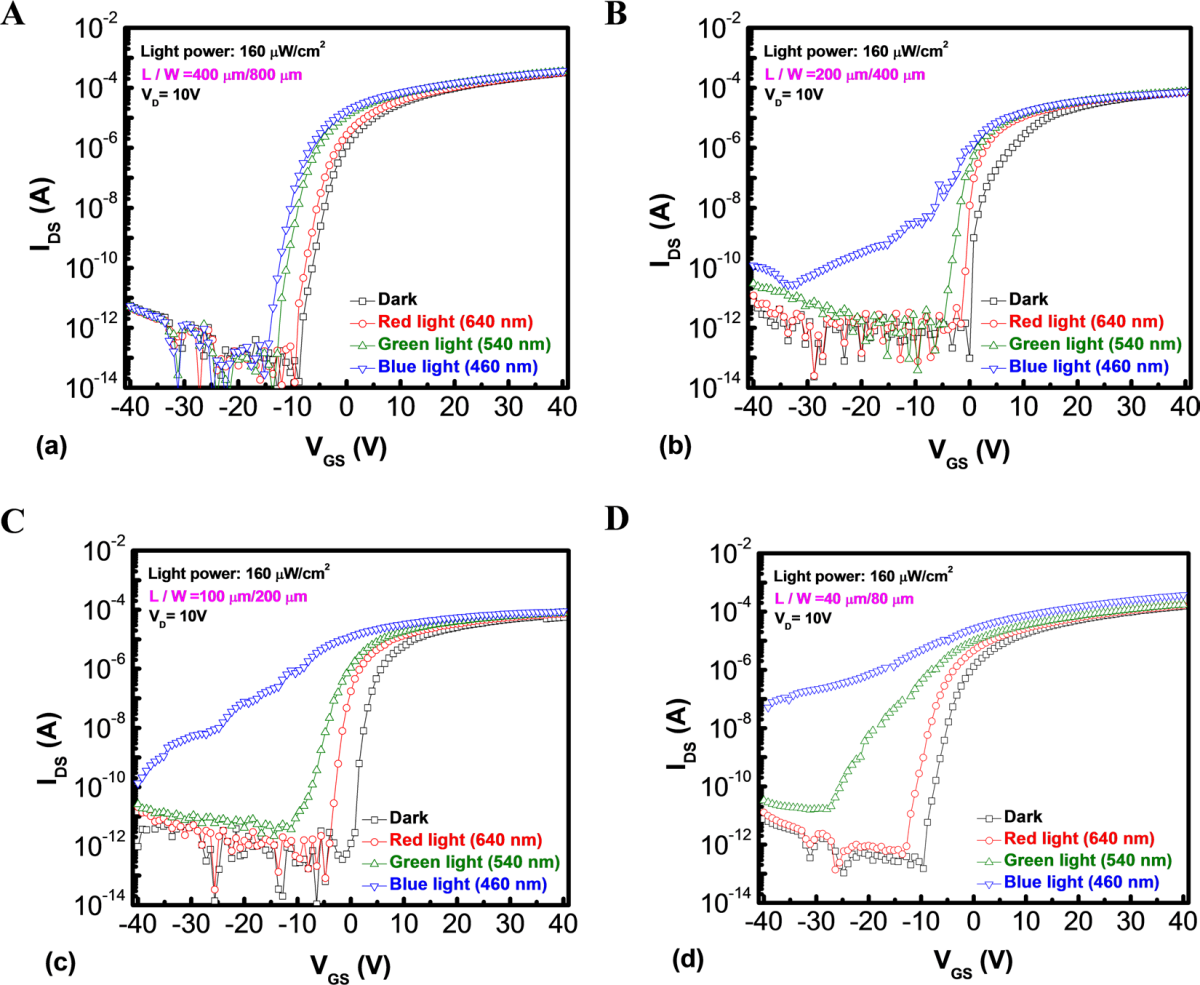
Transfer characteristics *I*_*DS*_*-V*_*GS*_ curves of a-IZO sensor TFT with a fixed ratio of channel length to width are compared in Fig. 2: (**A**) 400 μm/800 μm, (**B**) 200 μm/400 μm, (**C**) 100 μm/200 μm, and (**D**) 40 μm/80 μm, respectively, under the light wavelength of 640 nm (red light), 540 nm (green light) and 460 nm (blue light) illumination at a fixed light power of 160 μW/cm^2^.

**Figure 3:**
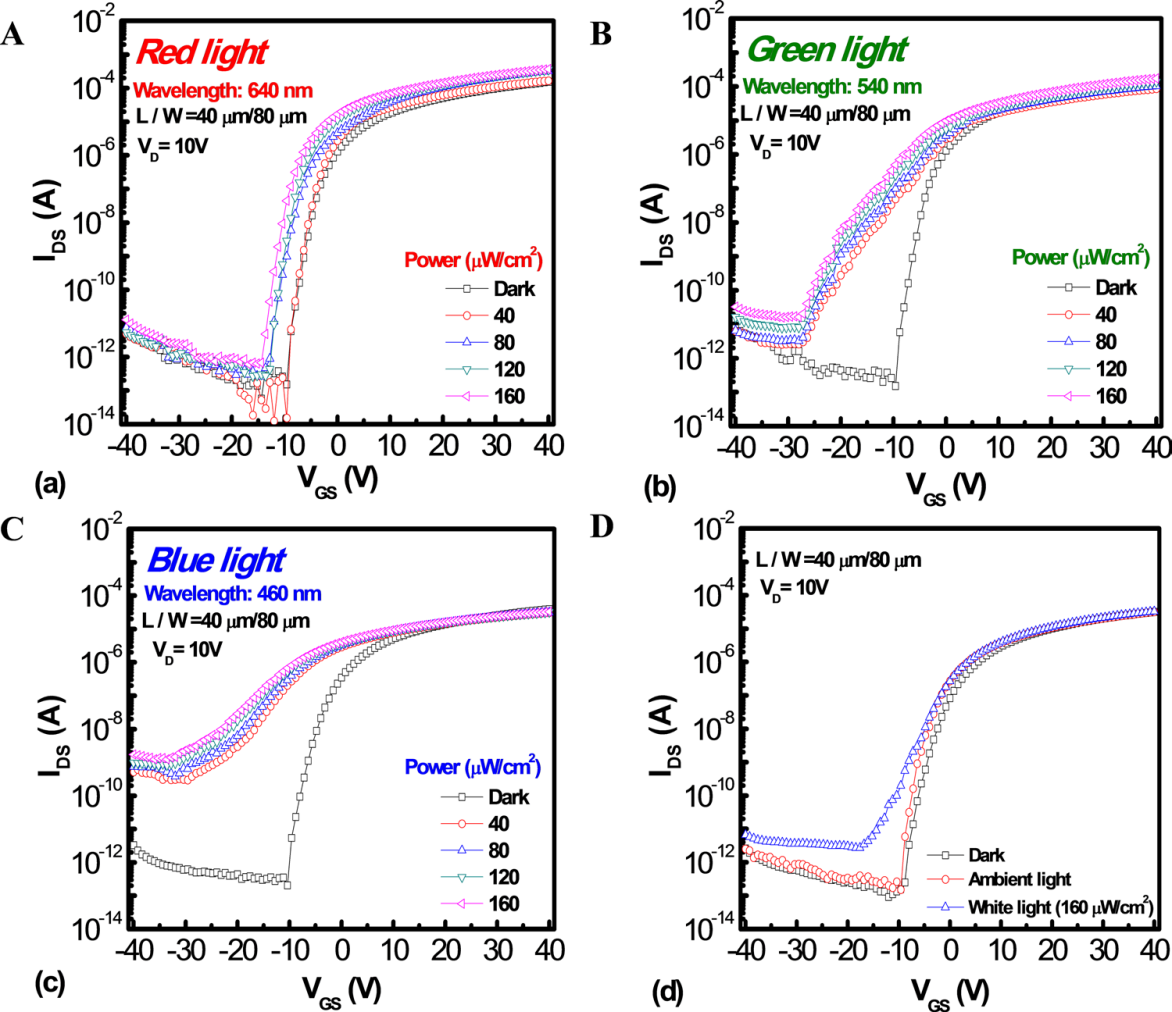
Transfer characteristics *I*_*DS*_*-V*_*GS*_ curves of a-IZO sensor TFT with the various light powers from initial to 160 μW/cm^2^ and light wavelength of (A) 640 nm (red light), (**B**) 540 nm (green light) and (**C**) 460 nm (blue light); (**D**)Transfer characteristics *I*_*DS*_*-V*_*GS*_ curves of a-IZO sensor TFT with different measure environment.

For remote sensing application, either light-induced drain current increasing in the off-state or a threshold voltage negative shift (*V*_*TH-Shift*_) can be used as sensing signal to check the power or the wavelength of light illumination. However, the signal of light-induced drain current is easier to detect and preferred to be applied on peripheral circuits of display panel than the signal of *V*_*TH-Shift*_^28^. In order to achieve the optimization of sensing characteristics, the *I*_*DS*_-*V*_*GS*_ curves of a-IZO sensor TFT with a fixed ratio of channel length to width are compared in Fig. 2: (a) 400 μm/800 μm, (b) 200 μm/400 μm, (c) 100 μm/200 μm, and (d) 40 μm/80 μm, respectively, under the light wavelength of 640 nm (red light), 540 nm (green light) and 460 nm (blue light) illumination at a fixed light power of 160 μW/cm^2^. It reveals that the off-state drain current exhibits an obvious change in the short-channel device than that of the long-channel one, especially under the blue light illumination. The off-state drain current is dominated by the light-induced current (*I*_*ph*_), which consists of excess carrier concentration (ionized oxygen vacancy) by the light-excited generation in the IZO channel layer^29^. On the contrary, *I*_*ph*_ of the IZO sensor under red light is very low. It suggests that the excited energy of light corresponded to the light wavelength is also important for the light-induced generation. In addition, there are apparent threshold voltage shifts for both samples under all the light illumination conditions. That means a higher excited energy may induce higher channel conductivity and a more negative gate bias is needed to deplete the excess carrier concentration. Then, the phenomenon should be common for the driver TFT. Therefore, the reliability characteristics of metal-capped driver IZO TFTs are also investigated in Figure S2 of Supplementary. On the other hand, with a combined effect of relatively high lateral field and the existence of V_o_^+^, both *E*_*c*_ and *E*_*v*_ for the short-channel device are bent downward and will form the conducting back channel from source to drain^30–32^. Under this hypothesis, the barrier lowering height of the short-channel device is more significant than that of the long-channel device, leading to anomalous high photocurrent for the short-channel device, which may be the reason for different results with various channel lengths. Then, the *I*_*DS*_-*V*_*GS*_ profiles of the short-channel a-IZO-based sensor TFT were measured under illumination conditions with various powers of light from an initial value to 160 *μ*W/cm^2^ and light wavelengths of (a) 640 nm (red light), (b) 540 nm (green light) and (c) 460 nm (blue light), respectively, as shown in Fig. 3. With a more negative gate bias applied, a higher *I*_*ph*_ can be observed. That is due to the accumulation of holes near the source electrode, which will enlarge the electron potential barrier for the carrier injection from the source to the channel region, contributing to a higher probability for light-excited generation. Afterwards, the device shows the best sensing performance under the wavelength of 460 nm (blue light and even UV). Furthermore, the *I*_*ph*_ exhibits a significant increase with the increasing light intensities of blue and green light illumination. As a result, the light intensity can be quantified by the output of off-state current. Under a precise quantitative and qualitative analysis by the way of systemic circuit design, the a-IZO sensor TFT can even simultaneously define both the wavelength and light intensity in a limit range of wavelengths and intensities for the blue light illumination. For example, several sensor device arrays with different channel lengths can exhibit different responsivity or signal to noise ratio for the same wavelength and intensity illumination. Thus, these features can be organized and used for signal correction and identification of light intensity and wavelength at the same time. If the sensing device is set to focus on the monochromatic light, like blue light for IZO film in this work, the system can be built up by countable correction sensing parts. On the other hand, more compensation circuit modules are needed to overcome the uniformity issues and temperature effects. Besides, the device was also measured under dark, ambient light, and white light illumination for comparison shown in Fig. 3(D). It suggests that the IZO material has a good ability to avoid the interference of ambient light, even for the white light. For further comparing the sensing characteristics, the spectral optical responsivity can be extracted from the *I*_*DS*_-*V*_*GS*_ graph, which is set at *V*_*GS*_ = −20 V and *V*_*DS*_ = 1 V under different wavelengths. It is calculated by the *I*_*ph*_ and incident light power, which refers to the device performance in terms of the photo gain of the device^33^.

1 $$R=\frac{{J}_{ph}}{{P}_{light}}=\frac{{I}_{ph}}{WL{P}_{light}},$$

In equation (1), *R* is the responsivity, *WL* is the area of the channel, *J*_*ph*_ is the photo current density, and *P*_*light*_ is the incident light power density. Besides, signal to noise ratio (*SNR*) is also a standard index to compare the level of a desired signal to the level of background noise, which is widely used in science and engineering^34^. The definition of *SNR* is described as following equation (2):

2 $$SNR=\frac{{I}_{ph}}{{I}_{dark}},$$

The *I*_*dark*_ is the initial off-state drain current without any illumination. Then, the spectral optical responsivity and *SNR* values of device under different wavelength of light are summarized in Table 1. In contrast, Table 2 exhibits the comparison of the device performance of the a-IZO sensor TFT in this work and some reported sensor TFT with different channel material under similar condition of blue light illumination in recent years^35–40^. The a-IZO sensor TFT shows a higher optical responsivity than other channel material in the recent investigation for its high density of oxygen vacancy, which is sensitive and easily ionized under the blue light illumination. In addition, the device uniformity is very important for controlling the system designed margin. All the uniformity data for both driver TFT and sensor TFT are compared in Figure S3 of Supplementary, while each error bar includes six different measuring results for each TFT devices.

**Table 1:** The optical responsivity and signal to noise ratio values of a-IZO sensor under different kinds of light source.

*V*_*G*_ = −20 *V*, *V*_*D*_ = 10 *V*		
Light source	Optical responsivity: R (A/W)	Signal to noise ratio (SNR)
ambient light	~0	similar to dark current
white light	~0	~10
red light, λ = 640(nm)	3.1*10^−4^	1~10
green light, λ=540(nm)	$1$.56	${10}^{4}$
blue light, λ=460(nm)	1280	10^5^

**Table 2:** Comparison of the device performance of the present a-IZO sensor TFT and some reported sensor TFT with different channel material under the blue light illumination.

	Wavelength (blue light)	Light power	Optical responsivity: R (A/W)	Signal to noise ratio (SNR)
a-Si:H TFT^35^	420 nm	160 μW/cm^2^	0.092	133
ZnO TFT^36^	430 nm	NA	0.026	213
a-MZO TFT^37^	450 nm	NA	0.47	10^5^
a-IGZO TFT^38^	430 nm	100 μW/cm^2^	Less than 0.5	10^4^
a-IGZO TFT^39^	450 nm	180 μW/cm^2^	NA	Less than 1
NW TFT^40^	500 nm	260 μW/cm^2^	NA	10^4^
This work	460 nm	160 μW/cm^2^	1280	10^5^

**Figure 4:**
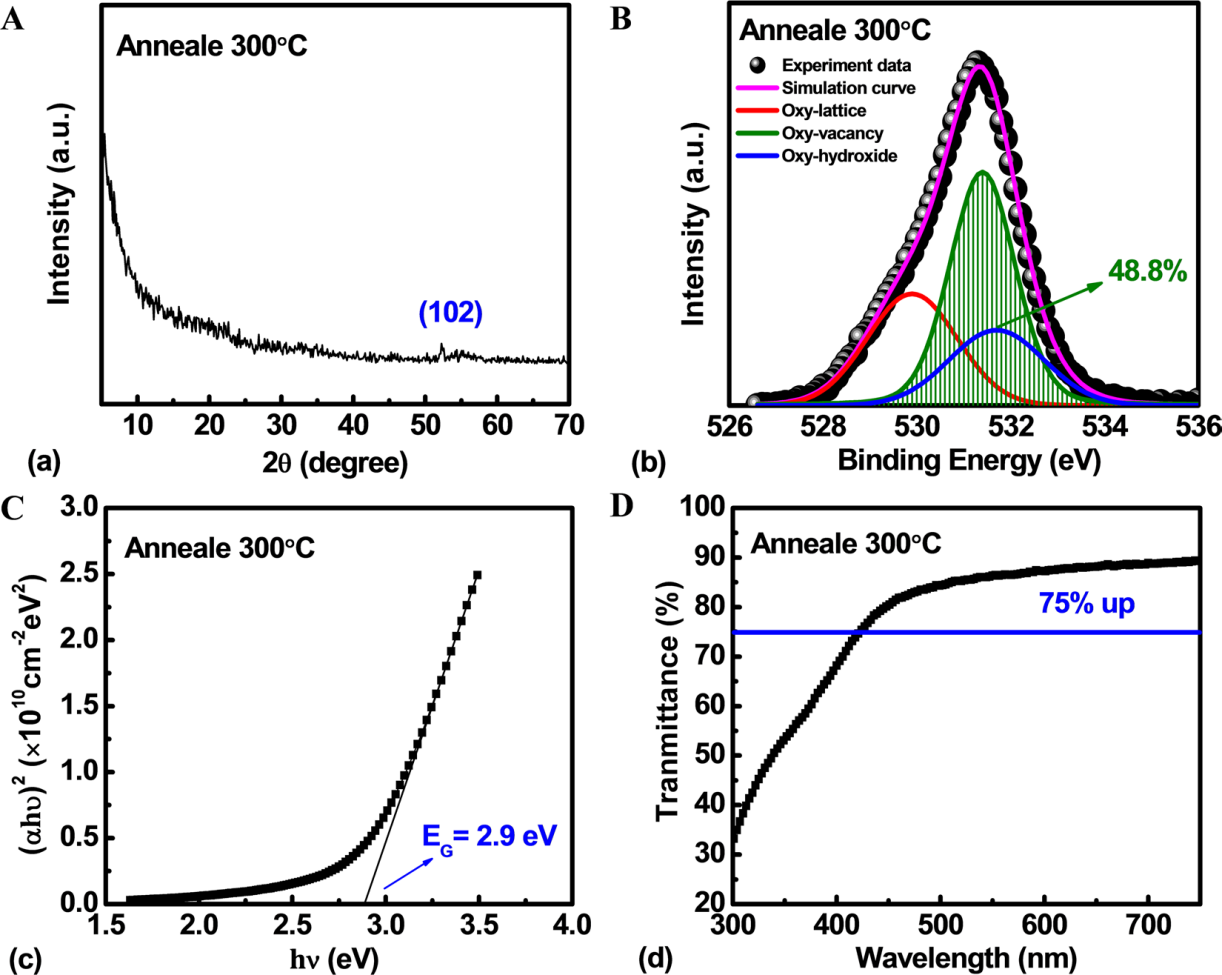
(**A**) X-ray Diffraction (XRD) spectra, (**B**) X-ray photo-electron spectroscopy (XPS), (**C**) Tauc plot, and (**D**) Transmittance spectra of a-IZO thin film treated with the same process of sensor TFT.

In order to further investigate material and optical characteristics of a-IZO thin film, X-ray Diffraction (XRD), X-ray photo-electron spectroscopy (XPS) and Ultraviolet-visible spectroscopy were used in this work. In the XRD pattern of IZO thin film shown in Fig. 4(A), there is not any sharp diffraction peak symbolizing the crystalline phase or poly phase. Therefore, the IZO thin film is considered as the amorphous phase treated by the same process with sensor device. Figure 4(B) shows the XPS spectra of O 1 s signal of IZO film. The O 1 s peaks centered at binding energies are 531.7 ± 0.25 eV, 531.4 ± 0.25 eV and 529.9 ± 0.25 eV related to oxygen in oxide lattices with OH^−^ impurities, with oxygen vacancies, and without oxygen vacancies (oxygen binding), respectively^41^. The relative area of the oxygen vacancy-related peak ratio is 48.8%, which is even higher than that of the oxygen lattice-related peak. It may be the reason for the absence of the carrier suppressor, like gallium element in a-IGZO channel, which may effectively lower the density of oxygen vacancy^42^. Hence, the IZO material shows a higher probability for ionizing the deep, neutral oxygen vacancy states to shallow donor states during the blue light illumination. On the other hand, the optical band-gap (*E*_*g*_) is about 2.9 eV, corresponded to the blue light wavelength of 430 nm, which can be determined from the linear fit of (*αhv*)^2^ against photo energy by applying the Tauc’s equation, as shown in Fig. 4(C). Furthermore, Fig. 4(D) shows the transmission spectra of the IZO thin film extracted by ultraviolet-visible spectroscopy. The transparent IZO film exhibits a good transmittance (over 75%) in visible region. Besides, the transmission spectra also proves that the wavelength of blue light and UV can be absorbed and detected effectively by the IZO film, which is desirable for the sensor device.

**Figure 5:**
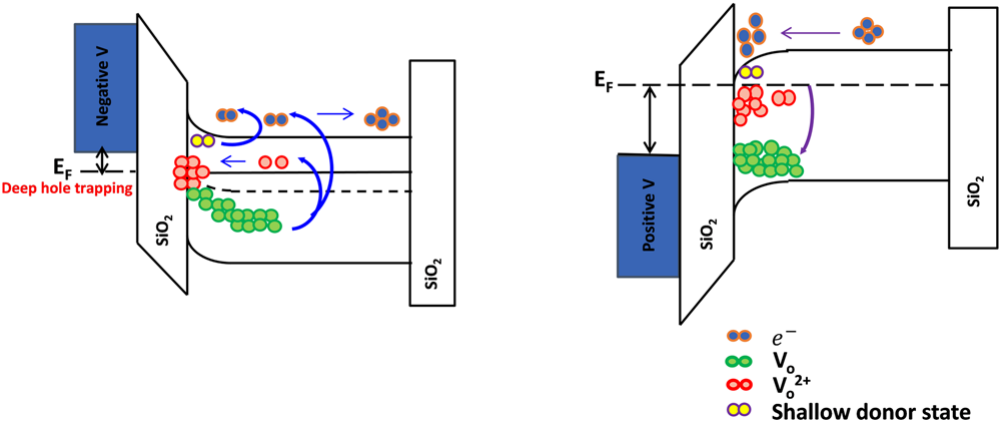
Schematic band diagram of the a-IZO sensor TFT, which conceptually depicts the mechanism of blue-light sensing and positive-bias-assisted PPC recovery.

**Figure 6:**
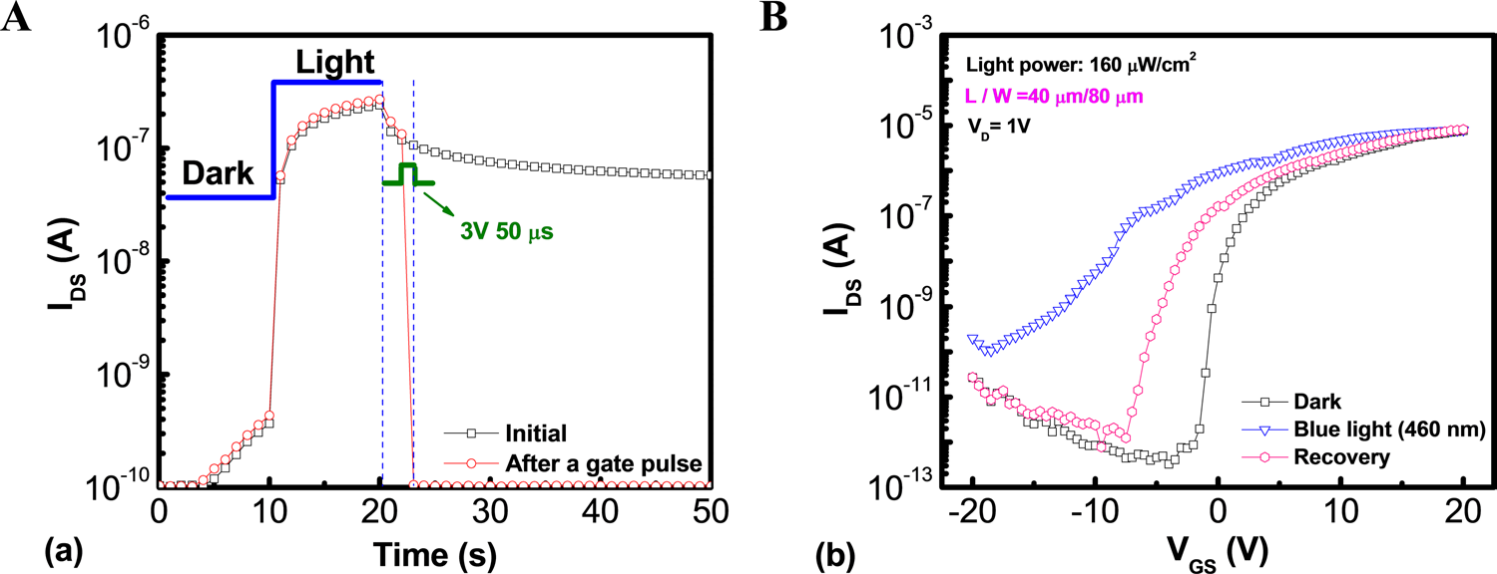
(**A**) I_DS_ versus measured time curves of a-IZO sensor TFT, while a 50 micro-seconds positive gate pulse was applied for recovering from PPC to the original dark state; (**B**) Transfer characteristics *I*_*DS*_*-V*_*GS*_ curves of a-IZO sensor TFT for the original dark state, during blue light illumination and after PPC recovery state.

Another restrict about the embedded sensor application may be the phenomenon of persistent photoconductivity (PPC)^43^, which can be induced by the remaining ionized oxygen defects even after illumination ceases and limit the frame-rate of active matrix imaging arrays in high-speed operation. Afterwards, a charge pumping technique was firstly proposed in our previous investigation and widely used in the academe to speed up the recoverability of electrical characteristic from the adverse effects of PPC^44^. Figure 5 shows the schematic band diagram of the a-IZO sensor TFT, which conceptually depicts the mechanism of blue-light sensing and positive-bias-assisted PPC recovery. Generally, the light-excited V_O_^2+^, which are generated from the sub-gap transition of neutral V_O_ by blue light illumination, would drift toward the gate insulator and be trapped as deep hole trapping centers in the band-gap under a negative gate bias. Meanwhile, the excess oxygen vacancies act as shallow donors and supply conduction electrons, inducing a narrower band-gap and resulting in a negative shift of the transfer curve and the climbing of photo-current. However, the negative gate bias will cause a physical separation of photo-generated electron-hole pairs by confining the electrons to the back channel, even after the illumination has stopped. As both the V_O_^2+^ sites and the photo-generated holes within the valence band are localized, physical confinement of the photo-generated electrons near the back channel further inhibits the recovery by slowing the reaction. This means that the recovery time is too long to be used as photo sensors without applying a positive gate bias. In this work, a 50 micro-seconds positive gate pulse was applied to sensor device (pulse height: 3 V; applied by an Agilent 81110 A pulse generator) to eliminate PPC as a verification depicted in Fig. 6(A). It reveals that an abrupt decrease of *I*_*ph*_ is observed after the PPC recovery. Besides, the transfer characteristic curves of a-IZO sensor TFT for the original dark state are shown in Fig. 6(B), measured at room temperature, during the blue light illumination and after the PPC recovery state. It also proves that the PPC can be overcome through deployment of a positive gate pulsing scheme within a few micro-seconds. However, the *V*_*TH*_ negative shift is still observed even after the pulse elimination process. It may be reason for that some excess carrier may be trapped in the channel material or gate insulator layer after charge pumping technique.

The effect of temperature is also investigated in this work. A higher dark state current and more negative *V*_*TH*_ shift occur with increasing measurement temperatures, indicating that the probability for ionizing the deep oxygen vacancy states into the shallow donor states will be higher at elevated temperatures. Figure S4 in the Supplementary section shows transfer characteristics of the a-IZO TFT sensor after blue light illumination and the positive gate pulse elimination under different measurement temperatures. Then, the phenomenon can be improved by a compensation circuit module, which can input the different data voltage to create corresponding negative *V*_*GS*_ for sensing unit and enhance the stability of system within different temperature environment, or just by reducing the thickness of channel layer for lowering the concentration of deep oxygen vacancy, while the responsivity may be slightly sacrificed.

**Figure 7:**
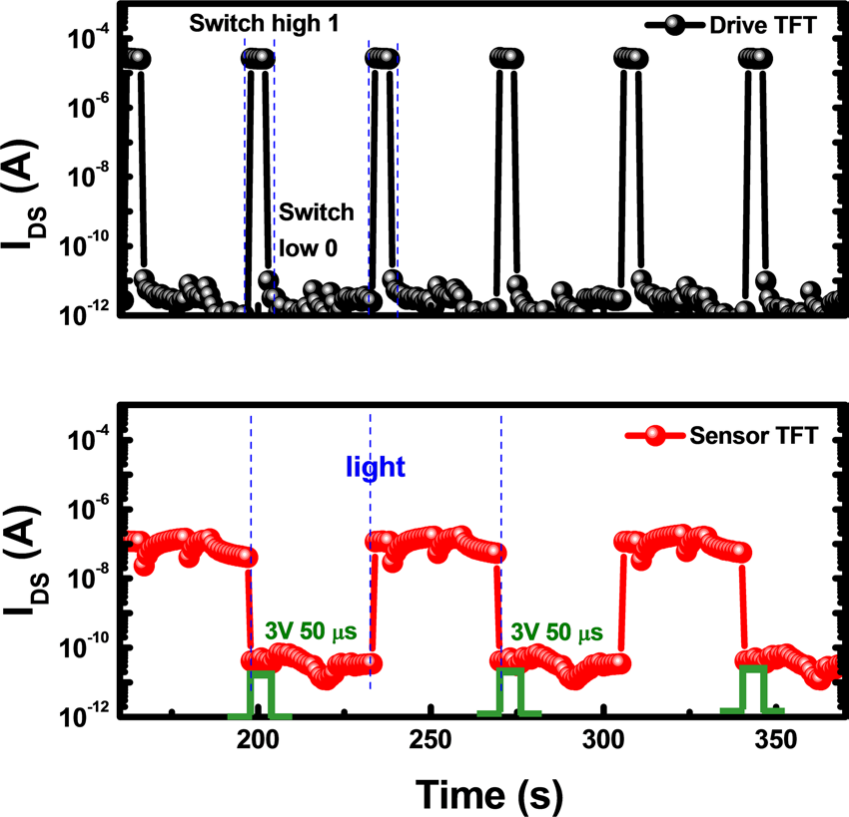
Evolution of *I*_*DS*_ versus measured time curves for the drive TFT and the sensor TFT integrated on the same substrate.

In order to confirm that the metal layer effectively shields the channel from illumination, the evolution of drain current (*I*_*DS*_) of both a-IZO TFTs (with and without light shielding metal layer device structure) were measured and shown in Fig. 7, which represents the driver TFT and sensor TFT, respectively. Afterwards, the measurement conditions of both a-IZO TFTs are list in Table 3. The device shows the same electrical property as switch characteristics even after light exposure because the light absorption in the active channel is effectively blocked by metal shielding layer, while the photo sensor TFT has an excellent response ability to light illumination and generates the photo current at the same time.

**Table 3:** The measurement setup of drive TFT with embedded optical sensor TFT.

	*V*_*G*_(V)	*V*_*D*_(V)	Period (sec)	Gate pulse (V, *μ* sec)
Drive TFT	20 (on) −20 (off)	5	40	NA
Sensor TFT	−20	5	80	3, 50

**Figure 8:**
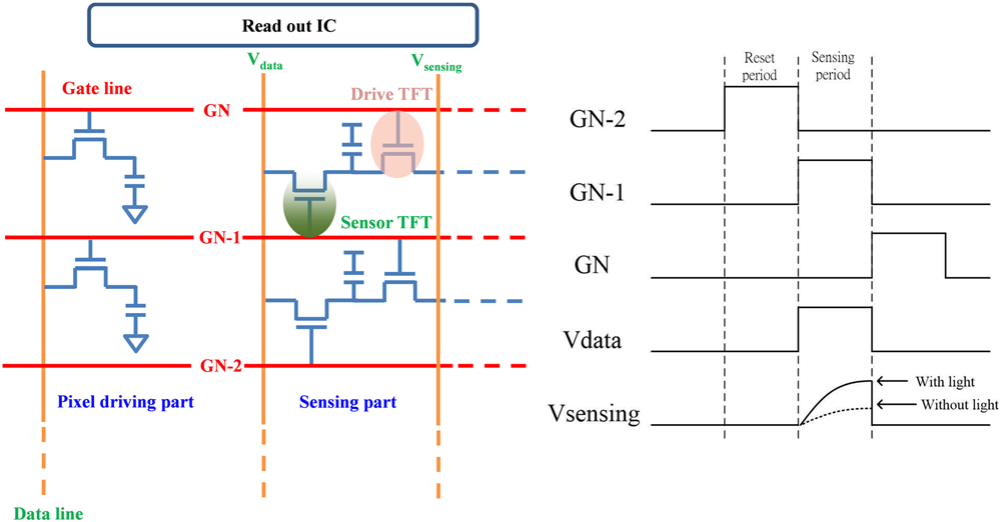
The schematic diagram of pixel circuit comprised of gate driver, source driver, photo sensing line, and read out integrated circuit (ROIC), while the time diagram is also included.

Figure 8 shows a schematic diagram of the pixel circuit embedded with photosensor and drivers, which is composed of gate driver, source driver, photo sensing line, and read out integrated circuit (ROIC), while the time diagram is included. It is designed that gate driver control both driving TFT and switching TFT, indicating that all steps to detect the state and visualize display are completed within single gate time, because the photo-current is large enough for ROIC to read the state for short time. In the sensing period, the gate line transports signal, and then the switch TFT turns on. With the light illumination, the sensor TFT on/off state detects the light, and the photo current transports to sensing line via a switch TFT. After that, the ROIC detects the sensing current and transports the signal to data line of driving pixel. The current through the driver TFT then displays the RGB pixel achieving the application of image sensor. For the reset period, the gate line provides gate pulse to reset sensor TFT, simultaneously, and the ROIC supplies the low voltage on data line of pixel driving to discharge the pixel capacitor.

In conclusion, an optical prototype of remote interactive panel is established to detect blue light by utilizing a-IZO TFT devices as both switching and sensor elements. Besides, the photo sensor a-IZO TFT shows both high optical responsivity and *SNR* ratio with excellent selectivity to wavelength and intensity. The PPC can be overcome through deployment of a gate pulsing scheme enabling realistic frame rates for advanced applications such as sensor-embedded display for touch-free interaction. At last, a sensor circuit integrated on RGB pixel with interactive display is also demonstrated on the basis of photo sensor TFT.

## Methods

Bottom gate staggered structure TFT devices were fabricated on heavily doped n-type Si (n^++^-Si) wafers with 100 nm-thick thermally grown oxide on top. An active (or sensing) layer of 20 nm-thick a-IZO film was deposited by RF reactive sputtering at room temperature in a mixture of argon (Ar), oxygen (O_2_) and nitrogen (N_2_) (Ar/O_2_/N_2_ = 27 sccm/2 sccm/1 sccm) using a target of In_2_O_3_:ZnO = 1:1 in mole ratio. Then, the source/drain electrodes were formed with RF-sputtered 50 nm-thick indium tin oxide (ITO). All the layers were patterned by photolithography using wet etching. The fixed ratio of channel length (L) and width (W) for the a-IZO TFTs were set at 400 *μ*m/800 *μ*m (long-channel length) and 40 *μ*m/80 *μ*m (short-channel length), respectively. In order to optimize the results of the embedded in-cell structure, the driver TFT devices were treated by N_2_O plasma treatment for 1 min, while the sensor TFT did not. Both the devices were sequentially capped with 100 nm SiO_2_ film as passivation layer deposited by plasma enhanced chemical vapor deposition (PECVD) in the mixture gases of SiH_4_/N_2_O and annealed by a horizontal furnace at 300 °C in the oxygen atmosphere. The basic electrical and sensing characteristics were measured in ambient air by using a Keithley 4200 semiconductor parameter analyzer with an optical fiber and a light source (STA-025) which can effectively control the wavelength and intensity of light. Furthermore, in order to investigate the PPC of a-IZO sensor, an ultra-fast sampling mode with 4225-Pulse Measurement Unit (PMU) module and K-Pulse pulse-generator-unit (PGU) was also applied during the blue light illumination. Besides, X-ray Diffraction (XRD), X-ray photo-electron spectroscopy (XPS) and Ultraviolet-visible spectroscopy were used for determining the material and optical characteristics of a-IZO thin film treated with the same process condition.

## Electronic supplementary material

Supplementary
