# Supplementary material for: Highly Responsive Blue Light Sensor with Amorphous Indium-Zinc-Oxide Thin-Film Transistor based Architecture
Source: Sci Rep. 2018 May 25;8:8153. doi: 10.1038/s41598-018-26580-5 (PMC5970141; doi:10.1038/s41598-018-26580-5)
Supplement: Supplementary file 1 — Supplementary [file 41598_2018_26580_MOESM1_ESM.doc]

Highly Responsive Blue Light Sensor with Amorphous Indium-Zinc-Oxide Thin-Film Transistor based Architecture

Po Tsun Liu^1*^, Dun Bao Ruan^2^, Xiu Yun Yeh^1^, Yu Chuan Chiu^1^, Guang Ting Zheng^1^, and Simon M. Sze ^2^

^1^Department of Photonics and Institute of Electro-Optical Engineering, National Chiao Tung University, Hsinchu 30010, Taiwan

^2^Department of Electronics Engineering, National Chiao Tung University, Hsinchu 30010, Taiwan

Correspondence and requests for materials should be addressed to Prof. Po-Tsun Liu (email: ptliu@mail.nctu.edu.tw, phone number: +886-3-5712121 ^#^ 52994)

In the main manuscript, a single layer of amorphous In-Zn-O thin film is chosen as the active layer material for both the drive device channel and photo sensing function. Meanwhile, with the detail material analysis and physical model, the photo sensing characteristics, channel length effect, and persistent photoconductivity of a-IZO sensor TFT are well discussed under blue light illumination. In addition, other important issues about this sensor TFT architecture like the commercial potentiality for scientific applications, the reliability of driver section, temperature effect of sensor section, and the uniformity characteristics are exhibited in this Supplementary.

**Supplementary Figures**

**Figure S1.** The future scientific applications of high performance blue light sensor, like self-piloting automobile under serious smog environment, fingerprint or precision image scanning, illumination self-adjustment of smart agriculture and biomedical electronic research.

**Figure S2.** *I_DS_-V_GS_* characteristics of metal-capped driver IZO TFTs (a) under positive gate bias stress, (b) under negative gate bias stress, and (c) under negative bias illumination stress.

**Figure S3.** The device parameter with error bar for (a) driver TFT, including the current ratio (*I_ON_/I_OFF_*), the field effect mobility (μ_FE_), and the threshold voltage (*V_TH_*); (b) sensor TFT, including optical responsivity, signal to noise ratio, and the current ratio (*I_ON_/I_OFF_*), each error bar includes six different measuring results for each TFT devices.

**Figure S4.** Transfer characteristics *I_DS_-V_GS_* curves of the a-IZO TFT sensor with dark state current, after blue light illumination, and after the positive gate pulse elimination under different measurement temperature; (a) 30 ^o^C, (b) 60 ^o^C, (c) 90 ^o^C, (d) 120 ^o^C, and (e) 150 ^o^C.
